# An Unusual Adenomatoid Tumor of Fimbria with Pronounced Psammoma Bodies in a BRCA Positive Patient as a Pitfall for Carcinoma on Frozen Section

**DOI:** 10.1155/2018/8148147

**Published:** 2018-11-21

**Authors:** Christine M. Lee, Michelle Moh, Peggy S. Sullivan, Neda A. Moatamed

**Affiliations:** Department of Pathology and Laboratory Medicine, David Geffen School of Medicine at UCLA, Los Angeles, CA, USA

## Abstract

**Background:**

BRCA gene mutations significantly increase the risk of breast and ovarian cancers where the lifetime risk of the ovarian cancer is about 40%. Therefore, many women with such mutations undergo prophylactic bilateral mastectomy and salpingo-oophorectomy. About 5-6% of these individuals display occult carcinomas in tubo-ovarian locations of which over 85% are tubal in origin. The objective of this case study was to emphasize emergence of benign lesions mimicking cancer under these circumstances.

**Case Report:**

We present a case with positive BRCA1 mutation who underwent the prophylactic procedure where a small mass was identified in her fallopian tube. Our initial encounter with this tumor was during intraoperative consultation. The tumor was associated with extensive psammoma bodies arranged in closely packed small tubules, mimicking serous carcinoma. Frozen section limitations including artifact, time constraint, and lack of ancillary studies as well as the clinical history further complicated our diagnostic assessment, which was deferred. A diagnosis of adenomatoid tumor was rendered on permanent sections.

**Conclusion:**

It is important to be familiar with this morphologic presentation of adenomatoid tumor as it is a pitfall for carcinoma, particularly on frozen section, and inaccurate diagnosis could lead to further unnecessary extensive procedures.

## 1. Background

BRCA gene mutations are a known risk factor for breast and ovarian cancers. The most common ovarian tumor in these patients is high-grade serous carcinoma which is believed to arise from a precursor lesion of fimbriae known as** s**erous** t**ubal** i**ntraepithelial** c**arcinoma (STIC) [1]. Women with BRCA mutations may undergo prophylactic bilateral mastectomy and salpingo-oophorectomy. Statistically around 5.4% of these asymptomatic cases have occult carcinomas discovered during the risk-reducing procedures and 86% of these lesions are tubal in origin [2].

Adenomatoid tumor is a benign neoplasm of mesothelial origin that can be seen in the female genital tract, more commonly involving the uterus and fallopian tubes than the ovary. It is the most common benign tumor of the fallopian tube [3]. Adenomatoid tumors pose a diagnostic challenge as they can histologically mimic malignant neoplasms, such as carcinoma, adenocarcinoma, mesothelioma, and Sertoli cell tumor [4]. Grossly, adenomatoid tumors are usually small (<2 cm), well-circumscribed, solitary masses that are tan-yellow, and solid [5]. Most are found incidentally. Microscopically, they are composed of cystic spaces or tubular gland-like structures lined by flattened to cuboidal mesothelial cells. Cells with intracytoplasmic vacuoles are also characteristic. Cytology is bland, and mitoses are absent [6].

The presence of psammoma bodies is notable in the gynecological tract. They are commonly associated with serous carcinoma though they can be seen in many different neoplasms [7]. In this study, we not only have described this unique histopathologic feature, but we also aimed to highlight the diagnostic challenges presented to us during both the frozen and permanent section evaluation of this tumor.

## 2. Case Report

The patient is a 61-year-old woman with BRCA1 gene mutation. Her medical history is significant for Cesarean section and left ovarian cystectomy. She does not have any history of cancer. She presented for prophylactic total laparoscopic hysterectomy and bilateral salpingo-oophorectomy. During the procedure, the surgeon discovered a white nodule on the fimbriated end of the right fallopian tube and sent the right salpingo-oophorectomy specimen for intraoperative consultation. He also noted adhesions between the omentum and the right pelvic sidewall. Otherwise, there were no other in situ or grossly visible abnormalities.

**Figure 1:**
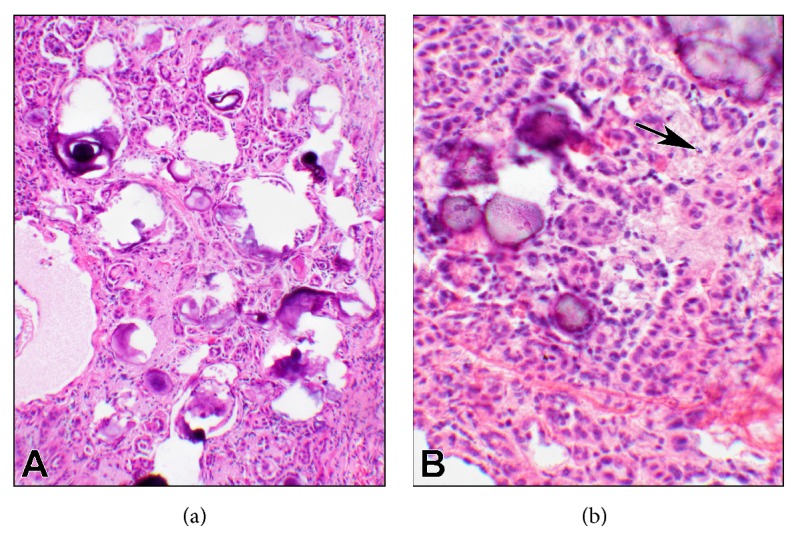
**Photomicrographs of the frozen section**. ((a), 10x objective) A low magnification of the lesion showing numerous psammoma bodies packed with the tubular nests. ((b), 20x objective) At a larger magnification, the psammoma bodies as well as the cellular morphologies are display with more details. Clusters of rather large cells with abundant foamy cytoplasm and eccentric nuclei were also present (arrow).

On gross examination, the nodule was solid and firm and measured 0.5 x 0.1 x 0.3 cm. The entire nodule was frozen, and sections showed small, tightly packed tubules and nests of epithelioid cells in a fibrous background notable for many psammoma bodies (Figure 1). The frozen section was interpreted as “epithelioid neoplasm with psammoma bodies, defer to permanents.”

**Figure 2:**
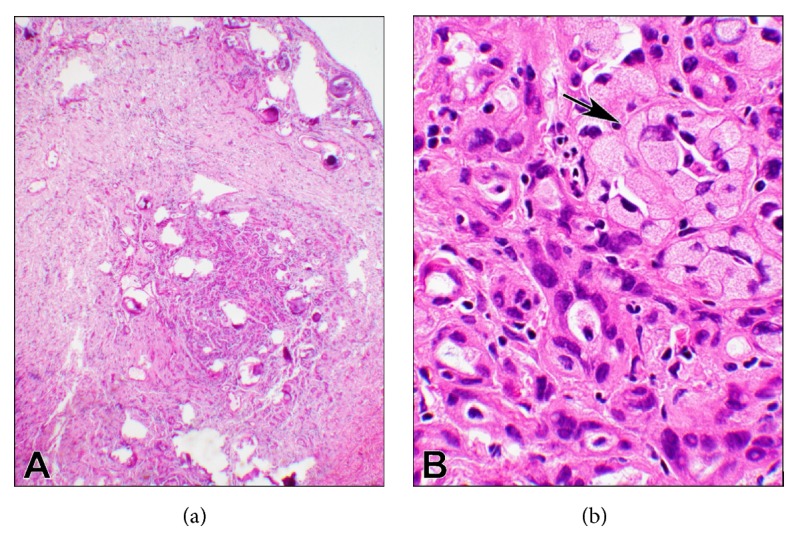
**Photomicrographs of the permanent section**. ((a), 4x objective) At a low magnification, the lesion shows a fibrous background with embedded tumor cells infiltrating the surrounding stroma. There are many psammoma bodies present throughout the tissue. ((b), 40x objective) At a higher magnification, there are tubular structures with hyperchromatic nuclei infiltrated by the inflammatory cells. Also, clusters of larger cells with foamy cytoplasm (arrow) are present.

**Figure 3:**
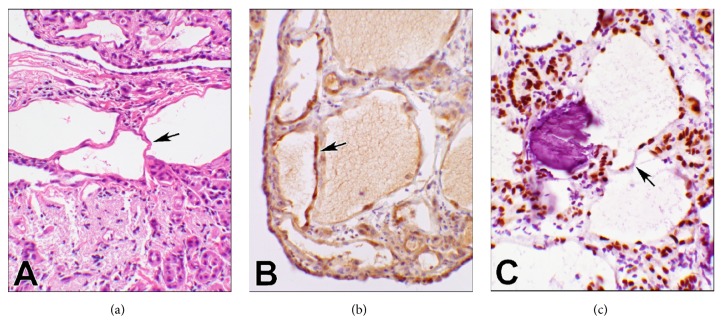
**Photomicrographs of thread-like bridging (**20x objective). The arrows point to the thread-like bridging feature of adenomatoid tumor in all three panels. (a) shows the bridges in the upper part and the solid tumor morphology in the lower segment of the photomicrograph stained with hematoxylin & eosin. (b) is calretinin IHC reaction of the cytoplasm and the nuclei, and (c) is PAX8 IHC reaction of the nuclei. Panel (c) has also captured a psammoma body.

On permanent section, a clearer cytologic assessment showed that the epithelioid cells were relatively small with regular, round nuclei, and even chromatin (Figure 2). There was no significant atypia. Mitoses were inconspicuous. The larger cells appeared similar to signet ring cells with a vacuolated cytoplasm. The psammoma bodies were a striking feature also on permanent section. Thread-like bridging, a typical feature of adenomatoid tumors [5], was observed in several foci of the lesion (Figures 3(a)–3(c)).

**Figure 4:**
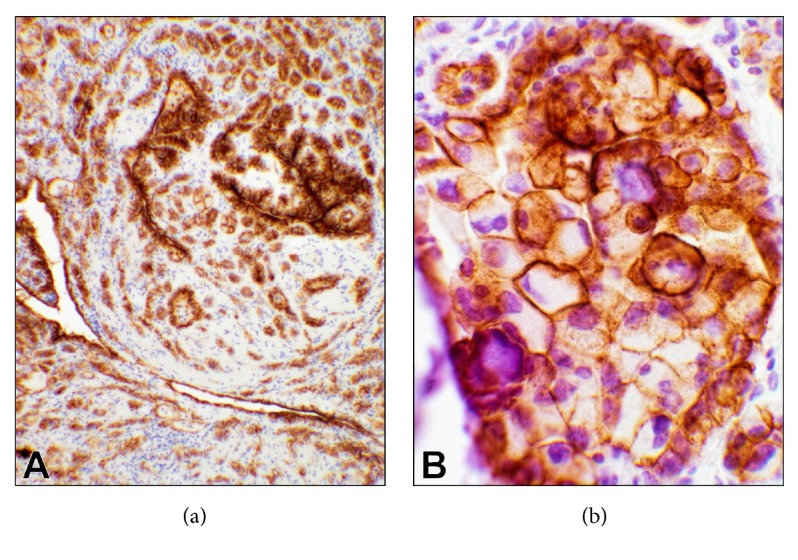
**Photomicrographs of D2-40 immunohistochemical (IHC) stain**. D2-40 IHC is a cell membrane stain and is a specific marker for mesothelial tumors as well as lymphoid endothelial cells and their neoplasms [8, 9]. ((a), 10x objective) The tumor cells as well as the vascular structures show strong reaction. ((b), 40x objective) A cluster of the foamy cells with the intense cell membrane reaction.

Ancillary studies were performed. Mucicarmine stain was performed to assess the presence of mucin in the vacuolated cells which was negative. Immunohistochemical (IHC) studies revealed positivity for WT1, calretinin (Figure 3(b)), PAX8 (Figure 3(c)), and D2-40 (Figure 4), and negativity for p53, estrogen-progesterone (ER-PR) receptors of the tumor cells. Epithelial markers including BerEP4/EpCAM, MOC-31, and B72.3 were all negative. The IHC reactions of WT1, calretinin, and D2-40 were positive and supportive of a mesothelial origin of the tumor [10, 11]. The vacuolated cells shared the same immunophenotype (Figure 4). Based on the histopathology and the IHC reactions, the lesion appeared benign and was diagnosed as adenomatoid tumor with psammoma bodies. The remaining fallopian tube and ovary, which were entirely submitted, did not reveal any other diagnostic abnormalities.

## 3. Discussion

Based on morphology alone, this case was difficult to distinguish from serous carcinoma, particularly at the time of intraoperative consultation. There were three major concerning morphologic features: (1) extensive psammoma bodies, which are typically associated with serous carcinoma; (2) tightly packed tubules and glands mimicking small solid nests on frozen section, a pattern commonly seen in low grade serous carcinoma; and (3) the presence of vacuolated cells, although a classic feature in adenomatoid tumors raised the question of signet ring cells and expanded the differential diagnoses to include mucinous adenocarcinoma. Furthermore, the patient's BRCA1 mutation status also increased the suspicion for a malignant process. In patients with BRCA mutations, the lifetime risk of ovarian cancer is as high as 40% whereas in the general population is only 1.4% [12]. Given these morphologic and clinical features, a definitive diagnosis could not be rendered during intraoperative consultation and was deferred. In such circumstances, it is entirely appropriate to give a descriptive interpretation and defer the diagnosis to permanent section evaluation.

The permanent sections provided an opportunity for a clearer histopathology observation. The lack of cytologic atypia and mitotic figures, two features commonly seen in serous carcinoma, lowered the suspicion for carcinoma at the time of surgery. However, since low grade serous carcinoma can have bland cytology and little to no mitotic activity [13], immunohistochemistry was essential to exclude the diagnosis of ovarian carcinoma and study other diagnostic possibilities. Lack of atypia and mitoses rules out the high-grade neoplasms. Negative epithelial markers, no expression of ER-PR receptors [14], and no staining of mucicarmine essentially exclude all ovarian carcinomas. On the other hand, positive calretinin and D2-40 in absence of p53 staining support the diagnosis of adenomatoid tumor [11]. Although positive, WT1 is the least reliable immunostain for mesothelial cells as serous carcinomas can also express the protein [13] whereas vacuolated cells and thread-like bridges are actually characteristic and supportive of the diagnosis of adenomatoid tumors [5, 15]. Positivity of PAX8 appears to be another unusual finding in this case. Generally, this marker is expressed in ovarian surface epithelial tumors as well as benign conditions such as endometriosis and endosalpingiosis [16]. However, aberrant PAX8 expression has been seen in well differentiated papillary mesothelioma, malignant mesothelioma of peritoneum, and rare cases of reactive mesothelial hyperplasia [17]. Although papillary mesothelial hyperplasia can show similar immunophenotypic expressions, this lesion is usually associated with other gynecological conditions, more commonly with endometriosis [4]. Our case did not have any other pathological lesions and was an isolated solitary nodule. In lieu of the gross and microscopic morphology including thread-like bridging (Figure 3) with consistent IHC findings, adenomatoid tumor is the best diagnostic fit for this lesion.

This unusual case highlights unique morphologic features seen in adenomatoid tumor that could be a diagnostic pitfall for carcinoma especially on frozen section accentuated by history of the BRCA mutation. An important lesson from this case is that the diagnosis should not be made during intraoperative consultation when the morphology does not align with that of a classic adenomatoid tumor and when the patient has a high clinical risk of malignancy. In these cases, morphologic evaluation on the permanent section may allow for better cytologic assessment, which proved to be crucial in our case. Also, special and immunohistochemical stains may be necessary to exclude carcinoma from the list of differential diagnoses.

It is noteworthy that psammoma bodies, in relation to female reproductive organs, may also be present in peritoneal mesothelial hyperplasia [4], cervical polyps, endometriosis, and endosalpingiosis [18]; in addition to the serous neoplastic lesions [19, 20]. Although presence of psammoma bodies appears to be rare in adenomatoid tumors of male and female reproductive organs, we failed to find any reference to that effect in our extensive search of PubMed and Google. Therefore, this case might be the first report of adenomatoid tumor associated with extensive psammoma body formation in the literature.

## 4. Conclusions

We hope pathologists will consider this presentation of adenomatoid tumor during diagnostic workup but maintain a high index of suspicion in the appropriate clinical and pathologic context. We emphasize that adenomatoid tumor is a benign lesion of the reproductive organs [3, 21] and can also occur in the patients with BRCA mutations.

## References

[1] Meserve E. E. K., Mirkovic J., Conner J. R. (2017). Frequency of “incidental” serous tubal intraepithelial carcinoma (STIC) in women without a history of or genetic risk factor for high-grade serous carcinoma: A six-year study. *Gynecologic Oncology*.

[2] Zakhour M., Danovitch Y., Lester J. (2016). Occult and subsequent cancer incidence following risk-reducing surgery in BRCA mutation carriers. *Gynecologic Oncology*.

[3] Hanada S., Okumura Y., Kaida K. (2003). Multicentric adenomatoid tumors involving uterus, ovary, and appendix. *Journal of Obstetrics and Gynaecology Research*.

[4] Oparka R., McCluggage W. G., Herrington C. S. (2011). Peritoneal mesothelial hyperplasia associated with gynaecological disease: A potential diagnostic pitfall that is commonly associated with endometriosis. *Journal of Clinical Pathology*.

[5] Hes O., Perez-Montiel D. M., Cabrero I. A. (2003). Thread-like bridging strands: a morphologic feature present in all adenomatoid tumors. *Annals of Diagnostic Pathology*.

[6] Sangoi A. R., McKenney J. K., Schwartz E. J., Rouse R. V., Longacre T. A. (2009). Adenomatoid tumors of the female and male genital tracts: a clinicopathological and immunohistochemical study of 44 cases. *Modern Pathology*.

[7] Das D. K. (2009). Psammoma body: A product of dystrophic calcification or of a biologically active process that aims at limiting the growth and spread of tumor?. *Diagnostic Cytopathology*.

[8] Arai E., Kuramochi A., Tsuchida T. (2006). Usefulness of D2-40 immunohistochemistry for differentiation between kaposiform hemangioendothelioma and tufted angioma. *Journal of Cutaneous Pathology*.

[9] Fukunaga M. (2005). Expression of D2-40 in lymphatic endothelium of normal tissues and in vascular tumours. *Histopathology*.

[10] Chu A. Y., Litzky L. A., Pasha T. L., Acs G., Zhang P. J. (2005). Utility of D2-40, a novel mesothelial marker, in the diagnosis of malignant mesothelioma. *Modern Pathology*.

[11] Hyun T. S., Barnes M., Laura Tabatabai Z. (2012). The diagnostic utility of D2-40, calretinin, CK5/6, desmin and MOC-31 in the differentiation of mesothelioma from adenocarcinoma in pleural effusion cytology. *Acta Cytologica*.

[12] Nebgen D. R., Hurteau J., Holman L. L. (2018). Bilateral salpingectomy with delayed oophorectomy for ovarian cancer risk reduction: A pilot study in women with BRCA1/2 mutations. *Gynecologic Oncology*.

[13] Boyd C., McCluggage W. G. (2012). Low-grade ovarian serous neoplasms (low-grade serous carcinoma and serous borderline tumor) associated with high-grade serous carcinoma or undifferentiated carcinoma: report of a series of cases of an unusual phenomenon. *The American Journal of Surgical Pathology*.

[14] Voutsadakis I. A. (2016). Hormone receptors in serous ovarian carcinoma: Prognosis, pathogenesis, and treatment considerations. *Clinical Medicine Insights: Oncology*.

[15] Phillips V., McCluggage W. G., Young R. H. (2007). Oxyphilic adenomatoid tumor of the ovary: A case report with discussion of the differential diagnosis of ovarian tumors with vacuoles and related spaces. *International Journal of Gynecological Pathology*.

[16] Laury A. R., Hornick J. L., Perets R. (2010). PAX8 reliably distinguishes ovarian serous tumors from malignant mesothelioma. *The American Journal of Surgical Pathology*.

[17] Xing D., Banet N., Sharma R., Vang R., Ronnett B. M., Illei P. B. (2018). Aberrant Pax-8 expression in well-differentiated papillary mesothelioma and malignant mesothelioma of the peritoneum: a clinicopathologic study. *Human Pathology*.

[18] Seguin R. E., Ingram K. (2000). Cervicovaginal psammoma bodies in endosalpingiosis: A case report. *The Journal of Reproductive Medicine*.

[19] Qazi F. M., Geisinger K. R., Barrett R. J., Hopkins 3rd M. B., Holleman Jr. I. L. (1988). Cervicovaginal psammoma bodies. The initial presentation of the ovarian borderline tumor. *Archives of Pathology and Laboratory Medicine*.

[20] Riboni F., Giana M., Piantanida P., Vigone A., Surico N., Boldorini R. (2010). Peritoneal psammocarcinoma diagnosed by a papanicolau smear: A case report. *Acta Cytologica*.

[21] Mhawech-Fauceglia P., Samroa D. (2018). A Case Report of an Adenomatoid Tumor of the Uterus Mimicking an Endometrioid Adenocarcinoma on Endometrial Curetting: a Diagnostic Pitfall. *Applied Immunohistochemistry & Molecular Morphology *.

